# Bufalin inhibits TGF-β-induced epithelial-to-mesenchymal transition and migration in human lung cancer A549 cells by downregulating TGF-β receptors

**DOI:** 10.3892/ijmm.2015.2268

**Published:** 2015-06-30

**Authors:** LEI ZHAO, SHIZHOU LIU, XIAOFANG CHE, KEZUO HOU, YANJU MA, CE LI, TI WEN, YIBO FAN, XUEJUN HU, YUNPENG LIU, XIUJUAN QU

**Affiliations:** 1 Department of Medical Oncology, The First Hospital of China Medical University, Shenyang, Liaoning 110001, P.R. China; 2 Department of Respiratory Medicine, The First Hospital of China Medical University, Shenyang, Liaoning 110001, P.R. China

**Keywords:** bufalin, lung cancer, transforming growth factor-β, receptor I, receptor II, epithelial-to-mesenchymal transition

## Abstract

The epithelial-to-mesenchymal transition (EMT) is a well-known prerequisite for cancer cells to acquire the migratory and invasive capacity, and to subsequently metastasize. Bufalin is one of the major active components of the traditional Chinese medicine Chan Su, and accumulating evidence has shown its anticancer effect in multipe types of cancer. However, the role of bufalin in transforming growth factor-β (TGF-β)-induced EMT and migration remains unclear. In the present study, the effect of bufalin on TGF-β-induced EMT and migration was investigated in human lung cancer A549 cells. TGF-β induced EMT in A549 cells and increased their migratory ability, which were markedly suppressed by bufalin. Additionally, TGF-β-induced upregulation of Twist2 and zinc finger E-box binding homeobox 2 (ZEB2), as well as the phosphorylation of Smad2 and Smad3 were also inhibited by bufalin. However, the Smad-independent signaling pathways were not affected. Further analysis showed that the TGF-β receptor I (TβRI) and TGF-β receptor II (TβRII) were downregulated in the presence of bufalin. Pretreatment with SB431542, a potent inhibitor of the phosphorylation of TβRI, significantly attenuated TGF-β-induced EMT, mimicking the effect of bufalin on A549 cells. Taken together, these results suggest that bufalin suppresses TGF-β-induced EMT and migration by downregulating TβRI and TβRII in A549 cells.

## Introduction

Lung cancer is one of the most common malignancies and major causes of cancer-related fatalities worldwide, and the majority of the patients with lung cancer present with advanced disease (1,2). Over the past few years, despite the significant advances that have been made in the treatment of advanced lung cancer, such as chemotherapy and targeted therapy, the majority of these patients succumb to cancer metastasis (3). Therefore, it is of importance to explore the underlying mechanisms of lung cancer metastasis.

Cancer metastasis is a complex process, during which the acquisition of migratory potential by cancer cells is a fundamental prerequisite (4). Epithelial-to-mesenchymal transition (EMT), a conversion in cell phenotype, has been recognized as one of the universal mechanisms by which cancer cells acquire the migratory and invasive capacities (5). During the process of EMT, epithelial cells acquire the fibroblastoid appearance due to downregulation of epithelial markers and upregulation of mesenchymal markers, thus, generating a migratory phenotype. Given the role of EMT in the onset of the metastatic cascade, controlling EMT is currently considered as a promising strategy to inhibit cancer metastasis and improve patient survival. However, the drug that can effectively block the occurrence of EMT has not been reported.

Bufalin is one of the main effective components of the traditional Chinese medicine Chan Su, which is obtained from the skin and parotid venom glands of the Chinese toad (6). Our previous study and others have shown that bufalin exerts anticancer effects by inducing cell cycle arrest, cell differentiation and cell apoptosis in various types of human cancer cells, such as leukemia, prostate, gastric, lung and hepatocellular carcinoma cells (7–14). Recently, several studies have suggested that bufalin inhibits cell migration, invasion and metastasis in several types of cancer, including hepatocellular carcinoma and osteosarcoma cells, partially through suppression of protein kinase B (AKT) and extracellular signal-regulated kinase (ERK), c-Jun N-terminal kinase (JNK) and p38 mitogen-activated protein (MAP) kinases signaling pathways (15–17). These signaling pathways are also involved in transforming growth factor-β (TGF-β)-induced EMT and migration (18). However, the effect of bufalin on EMT and migration of lung cancer cells mediated by TGF-β remains unclear.

In the present study, bufalin inhibits TGF-β-triggered EMT and the consequent cell migration of lung cancer A549 cells by downregulation of the TGF-β receptors, thus, providing novel evidence for its anticancer effect.

## Materials and methods

### Cell culture

The human lung cancer A549 cell line was purchased from the Type Culture Collection of the Chinese Academy of Sciences (Shanghai, China). The cells were cultured in RPMI-1640 medium (Gibco, Carlsbad, CA, USA) supplemented with 10% fetal bovine serum (FBS), penicillin (100 U/ml) and streptomycin (100 mg/ml) at 37°C, in a humidified incubator with 5% CO_2_.

### Reagents and antibodies

Recombinant human TGF-β was purchased from R&D Systems (Minneapolis, MN, USA). Bufalin and SB431542 were purchased from Sigma-Aldrich (St. Louis, MO, USA). Anti-E-cadherin (3195), anti-vimentin (5741), anti-phospho-Smad2 (Ser465/467; 3108), anti-Smad2 (5339), anti-phospho-AKT (Ser473; 9271), anti-AKT (9272), anti-phospho-p38 (Thr180/Tyr182; 9216), anti-p38 (9218), anti-phospho-JNK (Thr183/Tyr185; 9251), anti-JNK (9252), anti-TβRI (3712) and anti-TβRII (11888) antibodies were purchased from Cell Signalling Technology (Danvers, MA, USA). Anti-N-cadherin (ab12221), anti-fibronectin (ab6328), anti-Twist (ab50887), anti-Twist2 (ab57997), anti-Snail (ab135708), anti-Slug (ab27568), anti-phospho-Smad3 (Ser423/425; ab52903) and anti-Smad3 (ab28379) antibodies were purchased from Abcam (Cambridge, MA, USA). Anti-actin (sc-1616-R), anti-phospho-ERK1/2 (Thr202/Tyr204; sc-16982-R), anti-ERK1/2 (sc-154) and anti-zinc finger E-box binding homeobox 2 (ZEB2; sc-271984) antibodies were purchased from Santa Cruz Biotechnology, Inc. (Dallas, TX, USA).

### Cell viability assay

Cell viability was determined by the 3-(4,5-dimethylthiazol-2-yl)-2,5-diphenyltetrazolium bromide (MTT) assay. Cells were seeded at 2×10^4^ cells/well in 96-well plates and incubated overnight, and were treated with various concentrations of bufalin for 24 h. Subsequently, 20 *µ*l MTT (5 mg/ml) was added to each well and the cells were incubated for another 4 h at 37°C. Finally, the cells were lysed in 200 *µ*l dimethyl sulfoxide for 20 min at room temperature to solubilize the crystals, and the optical density (OD) was measured at 570 nm with a microplate reader (Bio-Rad Laboratories, Hercules, CA, USA). The experiment was performed three times and in triplicate.

### Flow cytometric analysis

Cells were seeded in 6-well plates and exposed to 5 ng/ml TGF-β alone or in combination with 50 nM bufalin for 24 h. The cells were collected and fixed with ice-cold 70% ethanol for 12 h, and subsequently incubated with 20 *µ*g/ml RNase A at 37°C for 30 min and 10 *µ*g/ml propidium iodide for 30 min in the dark. Finally, the samples were evaluated by flow cytometry and the data were analyzed using CellQuest software (Becton-Dickinson, San Jose, CA, USA).

### Western blot analysis

Cells were rinsed twice with phosphate-buffered saline (PBS) and lysed in 1% Triton lysis buffer [1% Triton X-100, 50 mM Tris-Cl (pH 7.4), 150 mM NaCl, 10 mM ethylene diaminete traacetic acid, 100 mM NaF, 1 mM Na_3_VO_4_, 1 mM phenylmethyl sulfonyl fluoride and 2 *µ*g/ml protinin] on ice. Subsequently, the protein concentrations were determined using the Lowry method. Total cell proteins (30–50 *µ*g) were separated by sodium dodecyl sulfate-polyacrylamide gel electrophoresis (SDS-PAGE) and electrophoretically transferred to nitrocellulose membranes (Millipore, Bedford, MA, USA). The membranes were blocked with 5% skimmed milk in Tris-buffered saline Tween-20 (TBST) buffer [10 mM Tris (pH 7.4), 150 mM NaCl and 0.1% Tween-20] for 2 h at room temperature and incubated with the primary antibodies at 4°C overnight. Subsequent to rinsing thoroughly with TBST buffer, the membrane was incubated with the corresponding horseradish peroxidase-conjugated secondary antibodies for 30 min at room temperature. Finally, following extensive rinsing with TBST buffer, proteins on the membranes were visualized by an enhanced chemiluminescence reagent (SuperSignal Western Pico Chemiluminescent substrate; Pierce, Rockford, IL, USA) in the Electrophoresis Gel Imaging analysis system (DNR Bio-Imaging Systems, Jerusalem, Israel).

### Immunofluorescence

The cells were seeded on coverslips, which were placed in the 6-well plate in advance. Following treatment with or without TGF-β (5 ng/ml) for 48 h, the cells were fixed with 4% paraformaldehyde for 15 min, permeabilized with 0.5% Triton X-100 for 10 min, blocked with 1% bovine serum albumin for 1 h at room temperature and incubated with anti-E-cadherin and anti-vimentin antibody at 4°C overnight. Subsequently, the cells were rinsed thoroughly with PBS, and were incubated with Alexa Fluor 546-conjugated goat anti-rabbit IgG (A-11010) or Alexa Fluor 488-conjugated goat anti-rabbit IgG (A-11034) (Molecular Probes, Eugene, OR, USA) for 1 h at room temperature in the dark. 4′,6-Diamidino-2-phenylindole (Sigma-Aldrich) was used to stain the nuclei for 5 min at room temperature. Following mounting with the antifade mounting medium (Beyotime Institute of Biotechnology, Haimen, China), the cells were visualized by fluorescence microscopy (BX60; Olympus, Tokyo, Japan).

### Wound healing assay

Cells were seeded in a 6-well plate and allowed to grow to nearly 100% confluence in culture medium. Subsequently, a cell-free line was manually created by scratching the confluent cell monolayers with a 200-*µ*l pipette tip. The wounded cell monolayers were washed three times with PBS and incubated in RPMI-1640 with 10% FBS containing 5 ng/ml TGF-β alone or in combination with 50 nM bufalin for 24 h. Five scratched fields were randomly chosen and the images were captured by bright-field microscope (IX51; Olympus). The percentage of wound closure was measured using Adobe Photoshop CS2 (Adobe Systems Inc., San Jose, CA, USA). The experiment was performed three times and in triplicate.

### Transwell migration assay

A 24-well chemotaxis chamber (8 nM pore size; Corning Inc., Corning, NY, USA) was used in the experiment. Briefly, 1×10^4^ cells in 200 *µ*l serum-free medium containing 5 ng/ml TGF-β alone or in combination with 50 nM bufalin were seeded in the upper chamber, and 500 *µ*l culture medium supplemented with 2.5% FBS was added to the bottom well. After incubation for 24 h, non-migrated cells were removed from the upper surface of the chamber with a wet cotton swab and cells on the lower surface of the chamber were stained using the Wright-Giemsa method. The migrated cells were counted in five random fields under bright-field microscope (DMI3000 B; Leica Microsystems, Wetzlar, Germany). The experiment was performed three times and in triplicate.

### Statistical analysis

All the statistical analyses were performed using the SPSS software (SPSS for Windows, version 16.0; SPSS, Inc., Chicago, IL, USA). Differences between two groups were evaluated by Student's t-test. A P-value <0.05 was considered to indicate a statistically significant difference.

## Results

### TGF-β induces EMT and promotes migration in A549 cells

**Figure 1:**
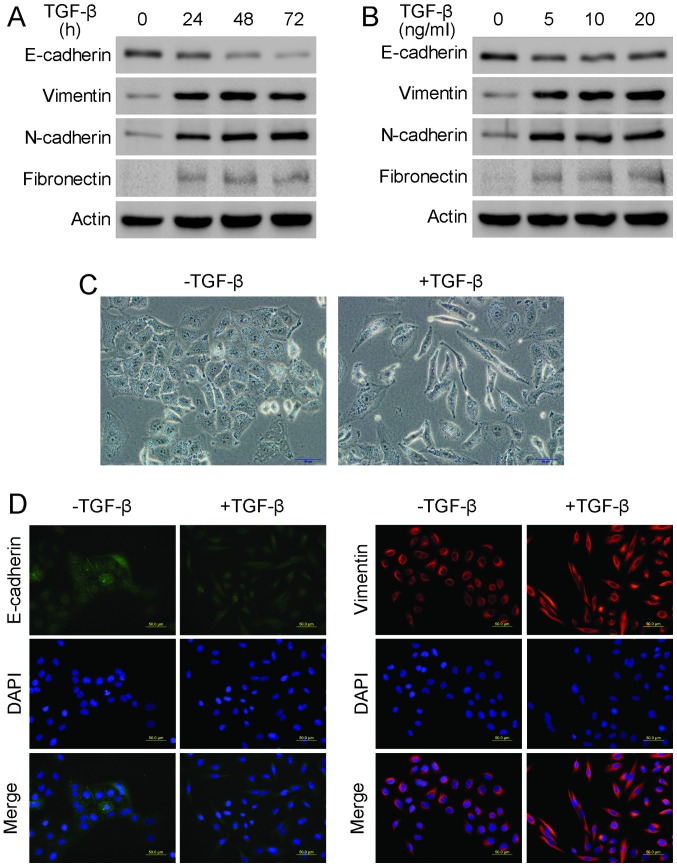
Transforming growth factor-β (TGF-β) induces epithelial-to-mesenchymal transition (EMT) in A549 cells. A549 cells were treated with 5 ng/ml TGF-β for the (A) indicated duration or (B) with various concentrations of TGF-β for 24 h. Subsequently, the epithelial and mesenchymal markers were analyzed by western blot analysis. After A549 cells were treated with 5 ng/ml TGF-β for 24 h, (C) the morphological changes were recorded by phase contrast microscopy (IX51; Olympus, Tokyo, Japan) (magnification, ×400) and (D) the changes in epithelial and mesenchymal markers in A549 cells were visualized by immunofluorescence microscopy (magnification, ×400). DAPI, 4′,6-diamidino-2-phenylindole.

**Figure 2:**
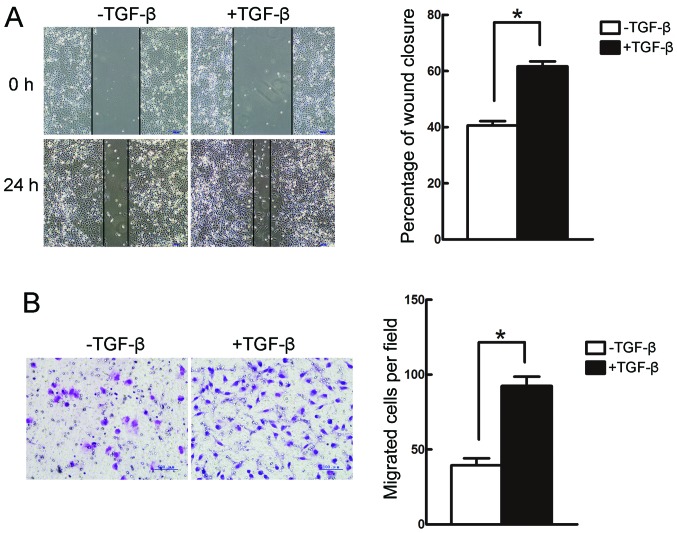
Transforming growth factor-β (TGF-β) promotes migration in A549 cells. A549 cells were treated with 5 ng/ml TGF-β for 24 h, and subsequently, the changes in migratory capacity were measured by the (A) wound healing assay (magnification, ×100) and (B) Transwell migration assay (magnification, ×200). The results are presented as the mean ± standard deviation, ^*^P<0.05.

To determine the appropriate concentration and duration for TGF-β to induce EMT, A549 cells were treated with 5 ng/ml TGF-β for the indicated durations or were incubated with various TGF-β concentrations for 24 h. Western blot analysis showed that the epithelial marker E-cadherin was downregulated and that the mesenchymal markers vimentin, N-cadherin and fibronectin were upregulated when the A549 cells were treated with 5 ng/ml TGF-β for 24 h, suggesting that EMT had occurred (Fig. 1A and B). Additionally, following treatment with 5 ng/ml TGF-β for 24 h, A549 cells underwent clear morphological changes, including disappearance of intercellular junction, cell elongation and spindle-like appearance, indicating that EMT had occurred (Fig. 1C). Additionally, the immunofluorescence assay showed that there was an evident decrease in E-cadherin and a significant increase in vimentin after the A549 cells were treated with 5 ng/ml TGF-β for 24 h, further confirming the occurrence of EMT in A549 cells (Fig. 1D). Furthermore, the wound healing and Transwell assays revealed that the migratory capacity of A549 cells was enhanced following incubation with TGF-β for 24 h (Fig. 2A and B). Therefore, treatment with 5 ng/ml TGF-β for 24 h was used in the following experiments.

### Bufalin suppresses TGF-β-induced EMT in A549 cells

**Figure 3:**
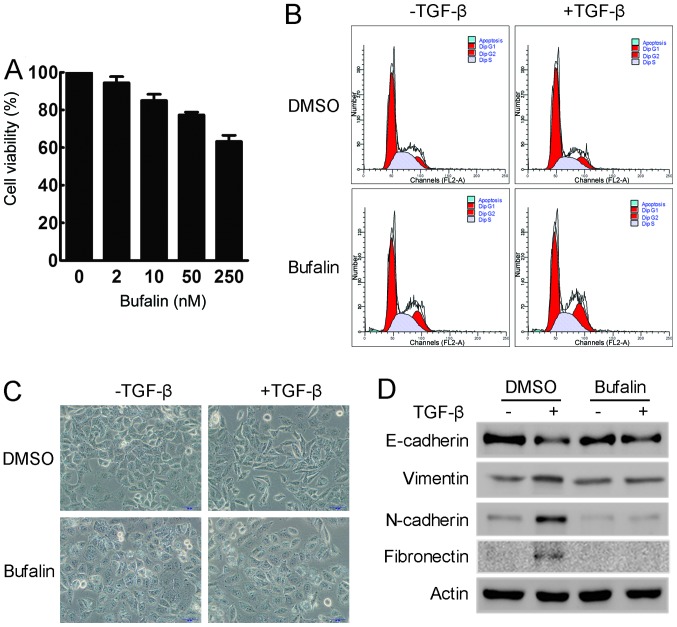
Bufalin inhibits transforming growth factor-β (TGF-β)-induced epithelial-to-mesenchymal transition (EMT). (A) After A549 cells were incubated with various concentrations of bufalin for 24 h, the cell viability was assessed by the MTT assay. The results are presented as the mean ± standard deviation. (B) A549 cells were treated with 50 nM bufalin for 24 h and stained with propidium iodide, and subsequently, cell cycle distribution and apoptosis were evaluated by flow cytometry. After A549 cells were treated with 5 ng/ml TGF-β alone or in combination with 50 nM bufalin for 24 h, (C) the morphological changes were captured by phase contrast microscopy (magnification, ×400), and (D) the expression of epithelial and mesenchymal markers was examined by western blot analysis. DMSO, dimethyl sulfoxide.

Since bufalin is mainly known as a cytotoxic agent, the effect of bufalin on cell viability was examined. A549 cells were treated with various concentrations of bufalin for 24 h. The MTT assay showed that incubation with 50 nM bufalin for 24 h did not significantly suppress the cell viability of A549 cells (Fig. 3A). Additionally, as shown by flow cytometry, treatment with 50 nM bufalin for 24 h had minimal effect on the cell cycle distribution of A549 cells and did not induce apoptosis in A549 cells (Fig. 3B). Thus, 50 nM bufalin was used in the following experiments.

To determine the effect of bufalin during TGF-β-induced EMT, the morphological changes in A549 cells treated with TGF-β alone or in combination with bufalin were examined. Treatment with TGF-β induced prominent morphological changes in A549 cells, including cell elongation and spindle-like appearance, indicating that A549 cells had undergone EMT. These changes were clearly inhibited by concomitant treatment with bufalin, as evidenced by a decrease in elongated and spindle-like cells (Fig. 3C). In addition, western blot analysis showed that the expression of epithelial markers, such as E-cadherin, was significantly reduced, while that of mesenchymal markers, such as vimentin, N-cadherin and fibronectin, was increased following incubation with TGF-β. However, simultaneous treatment with bufalin suppressed all these changes (Fig. 3D). These data suggest that bufalin can effectively inhibit TGF-β-induced EMT in A549 cells.

### Bufalin inhibits TGF-β-induced migration in A549 cells

**Figure 4:**
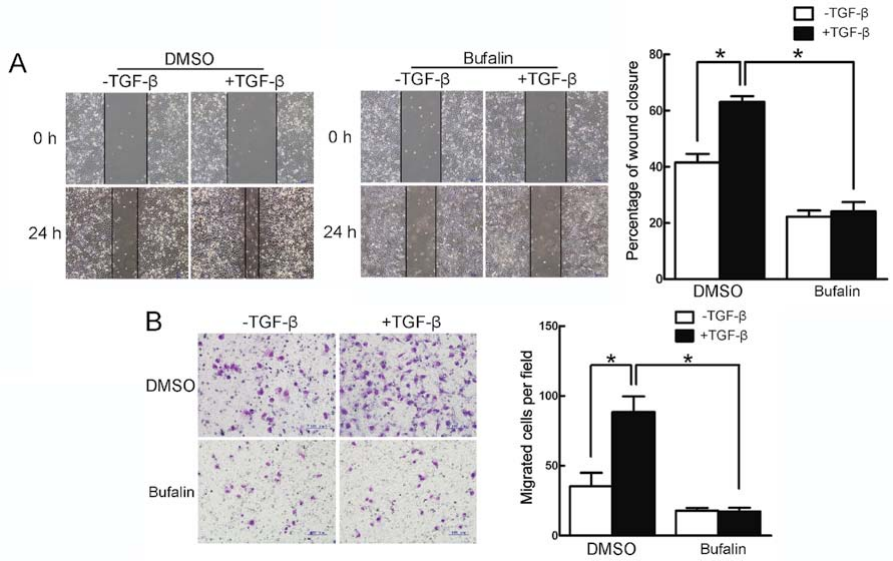
Bufalin supresses transforming growth factor-β (TGF-β)-mediated migration. A549 cells were treated with 5 ng/ml TGF-β alone or concomitantly with 50 nM bufalin for 24 h, and subsequently, the changes in migratory capacity were measured by the (A) wound healing assay (magnification, ×100) and (B) Tran-swell migration assay (magnification, ×200). In the assays, the results are presented as the mean ± standard deviation, ^*^P<0.05. DMSO, dimethyl sulfoxide.

The effect of bufalin on TGF-β-induced migration in A549 cells was further investigated by the wound healing and Transwell assays. In the wound healing assay, TGF-β facilitated the closure of the scratched area on the cell monolayers, which was inhibited by bufalin (Fig. 4A). The Transwell assay showed that TGF-β significantly increased the cells that migrated to the lower side of the filter, whereas concomitant incubation with bufalin evidently suppressed the TGF-β-induced increase in migrated cells (Fig. 4B). Thus, these findings demonstrate that TGF-β-induced migration in A549 cells is efficiently suppressed by bufalin.

### Bufalin inhibits TGF-β-induced upregulation of transcripton factors via downregulating TGF-β receptors

**Figure 5:**
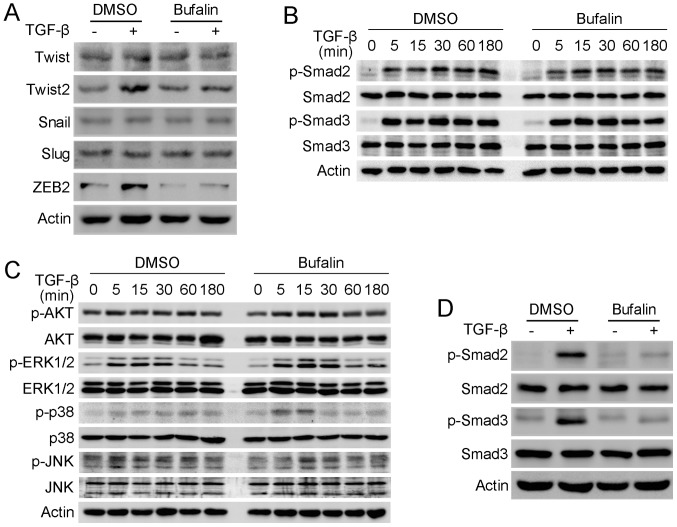
Bufalin inhibits transforming growth factor-β (TGF-β)-induced upregulation of transcription factors and activation of Smad signaling. After A549 cells were exposed to 5 ng/ml TGF-β alone or simultaneously with 50 nM bufalin for 24 h, (A) the expression of EMT-related transcription factors and (D) the phosphorylation of receptor-activated Smad proteins were analyzed by western blot analysis. A549 cells were treated with 5 ng/ml TGF-β alone or simultaneously with 50 nM bufalin for indicated duration, and subsequently, the phosphorylation of (B) receptor-activated Smad proteins and (C) Smad-independent pathway proteins were detected by western blot analysis. DMSO, dimethyl sulfoxide.

**Figure 6:**
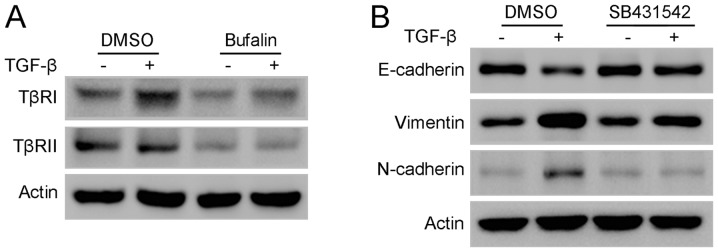
Bufalin downregulates transforming growth factor-β receptor I (TβRI) and TβRII. (A) After A549 cells were subjected to 5 ng/ml TGF-β alone or concomitantly with 50 nM bufalin for 24 h, the changes of TGF-β receptors were analyzed by western blot analysis. (B) A549 cells, pretreated with or without 10 *µ*M SB431542 for 2 h, were incubated with 5 ng/ml TGF-β for 24 h, and the expression of epithelial and mesenchymal markers was detected by western blot analysis. DMSO, dimethyl sulfoxide.

In order to reveal the mechanism by which bufalin inhibits TGF-β-mediated EMT and migration in A549 cells, the changes in EMT-related transcription factors induced by bufalin were investigated. Western blot analysis showed that TGF-β induced the upregulation of Twist2 and ZEB2, but not Twist, Snail or Slug in the A549 cells, and the TGF-β-induced-upregulation of Twist2 and ZEB2 was prominantly suppressed when A549 cells were concomitantly treated with TGF-β and bufalin (Fig. 5A). As TGF-β functions mainly through Smad and Smad-independent signaling pathways, the change of these signaling pathways was investigated. Smad and Smad-independent signaling pathways were not inhibited following treatment with bufalin for 3 h. The phosphorylation of AKT, ERK, p38 and JNK MAP kinases terminated within 1 h, while the phosphorylation of Smad2 and Smad3 continued until 3 h (Fig. 5B and C). Subsequently, the effect of bufalin on the phosphorylation of Smad2 and Smad3 was further examined after A549 cells were treated for 24 h. Western blot analysis demonstrated that bufalin inhibited the phosphorylation of Smad2 and Smad3 activated by TGF-β after incubation with bufalin for 24 h, while their total protein levels remained unchanged (Fig. 5D). As Smad2 and Smad3 are directly activated by TGF-β receptors, the changes of TGF-β receptor I (TβRI) and TGF-β receptor II (TβRII) were studied further. Western blot analysis showed that TβRI and TβRII were significantly downregulated following treatment with bufalin for 24 h (Fig. 6A). In addition, SB431542, a potent inhibitor of the phosphorylation of TβRI, significantly attenuated TGF-β-stimulated EMT in A549 cells, mimicking the effect of bufalin on A549 cells (Fig. 6B). Taken together, these results indicate that bufalin suppresses TGF-β-induced EMT and migration in A549 cells by downregulating TGF-β receptors.

## Discussion

As EMT promotes cancer cell metastasis, its mechanism has been studied extensively. EMT can be induced by numerous cytokines and growth factors, particularly TGF-β (19). TGF-β is a pleiotropic cytokine that regulates various biological processes, such as embryogenesis, adult tissue homeostasis, fibrosis and cancer progression (20). TGF-β binds to TβRII on the cell surface, resulting in the assembly of a hetero-tetrameric receptor complex, in which TβRII phosphorylates and activates TβRI. Activated TβRI phosphorylates the receptor-activated Smad proteins (R-Smads), mainly Smad2 and Smad3, which translocate into the nucleus and bind to DNA with transcriptional coactivators or corepressors to control the expression of target genes (20). Additionally, TGF-β also activates Smad-independent signaling pathways, such as AKT, ERK, JNK and p38 MAP kinases to exert its diverse function (21). Several studies have shown that TGF-β induces EMT and enhances the migratory capacity in different cell types (22–24). In the present study, TGF-β stimulates morphological changes, characteristic of EMT, in a time-dependent manner accompanied by downregulation of E-cadherin and upregulation of vimentin, N-cadherin and fibronectin in A549 cells. In addition, the present results demonstrate that TGF-β significantly enhances the migratory potential of A549 cells as a result of EMT.

Bufalin has been shown to inhibit cell migration in certain types of cancer cells (17,25). To the best of our knowledge, the present study shows for the first time that bufalin effectively suppresses TGF-β-induced-EMT and migration in A549 cells. Transcription factors, such as Twist, Twist2, Snail, Slug and ZEB2, repress the expression of E-cadherin and have a key role in EMT and migration (26–30). Additionally, Smad and Smad-independent signaling pathways are involved in TGF-β-induced EMT and upreguation of EMT-related transcription factors (18). Furthermore, studies have established the indispensable role of Smad signaling, mainly Smad2 and Smad3, in TGF-β-stimulated EMT (31,32). Smad signaling initiates Twist2 and ZEB2 transcription following activation by TGF-β (33,34). The present results show that bufalin significantly inhibits the upregulation of Twist2 and ZEB2, but does not affect Smad-independent signaling pathways, including AKT, ERK, p38 and JNK MAP kinases, during TGF-β-mediated EMT in A549 cells. By contrast, the TGF-β-induced phosphorylation of Smad2 and Smad3 is significantly suppressed following treatment with bufalin for 24 h. This may help the understanding of why the upregulation of Twist2 and ZEB2 during TGF-β-induced EMT is inhibited by bufalin. Smad2 and Smad3 are directly phosphorylated by TβRI, which is phosphorylated and activated by TGF-β-bound TβRII (20). Numerous studies have suggested that TGF-β signaling is precisely controlled through the modulation of TGF-β receptors (35–37). However, the detailed mechanisms of the regulation of TGF-β receptors by bufalin remain unclear. The present results show that TβRI and TβRII are significantly downregulated following treatment of A549 cells with bufalin, and that SB43152, the specific inhibitor of the phosphorylation of TβRI, has a similar effect to bufalin on A549 cells. Thus, TGF-β receptors may be the target for bufalin to inhibit TGF-β signaling.

In conclusion, the present results indicate that bufalin suppresses TGF-β-induced EMT and migratory capacity in human lung cancer A549 cells through downregulating TGF-β receptors. These findings warrant further assessment of bufalin in clinically relevant models to explore its potential role in the treatment of metastatic lung cancer.
